# The Influence of Alternative Diets and Whole Dry Black Soldier Fly Larvae (*Hermetia illucens*) on the Production Performance, Blood Status, and Egg Quality of Laying Hens

**DOI:** 10.3390/ani14172550

**Published:** 2024-09-02

**Authors:** Ana Montalbán, Josefa Madrid, Fuensanta Hernández, Achille Schiavone, Eduardo Ruiz, Cristian J. Sánchez, Lucía Ayala, Edoardo Fiorilla, Silvia Martínez-Miró

**Affiliations:** 1 Department of Animal Production, Faculty of Veterinary Science, Regional Campus of International Excellence “Mare Nostrum”, University of Murcia, Espinardo, 30100 Murcia, Spain; ana.montalban1@um.es (A.M.); nutri@um.es (F.H.); eduardo.ruizh@um.es (E.R.); cristianjesus.sanchez@um.es (C.J.S.); lucia.ayalag@um.es (L.A.); silviamm@um.es (S.M.-M.); 2 Department of Veterinary Sciences, University of Turin, 10124 Turin, Italy; achille.schiavone@unito.it (A.S.); edoardo.fiorilla@unito.it (E.F.)

**Keywords:** dry larvae, black soldier fly, layers, poultry, egg quality, performance, production, biochemical profile, blood traits, soybean meal

## Abstract

**Simple Summary:**

The European Union’s poultry industry relies heavily on imported ingredients such as soybean meal. In this study, we tested three diets on 120 laying hens over 15 weeks: a soybean–corn diet, an alternative diet with locally sourced plant-based ingredients (peas, distillers’ dried grains with solubles, and sunflower meal), and this alternative feed supplemented with 5% whole dried black soldier fly larvae. The results suggest that a novel alternative diet using fewer conventional ingredients could be developed into a flexible formulation that ensures productivity, sustainability, egg quality, and hen health.

**Abstract:**

Given the significant environmental consequences of current poultry feed practices and the heavy dependence of the European Union on imported soybeans, studying alternatives is crucial. This study evaluated the potential benefits of using locally sourced alternative plant-based ingredients and whole dry black soldier fly larvae in the diet of laying hens. The experiment involved 120 Isazul hens at 23 weeks old, which were divided into three groups with five replicates each (eight hens per replicate): a control diet (CON) based on soybean meal and cereals, an alternative diet (ALT) replacing the soybean meal with locally sourced plant-based resources (peas, distillers’ dried grains with solubles, and sunflower meal), and the ALT diet supplemented with 5% whole dry black soldier fly larvae (ALT+DBSFL). Over 15 weeks, the hens were fed ad libitum, and the production performance, egg quality, and plasma biochemical parameters were assessed across three experimental sub-periods. The results showed no significant differences in body weight, feed intake, egg production, egg weight, egg mass, or feed conversion ratio across the diets (*p* > 0.05). The egg quality remained consistent across all the groups; however, the hens fed the ALT+DBSFL diet exhibited higher Haugh units in the first experimental sub-period (*p* < 0.05) and lower plasma cholesterol and triglycerides at 32 weeks of age (*p* < 0.05). The findings of this study indicate that incorporating these alternative ingredients and whole DBSFL into layers’ diets does not compromise production performance, egg quality, or biochemical parameters related to health status, supporting their potential as sustainable feed alternatives.

## 1. Introduction

Numerous studies have indicated that feeding is the factor with the most substantial environmental impact in the poultry sector, accounting for up to 82% of the total impact of climate change, supporting the theory that adopting environmentally friendly feeding practices has the potential to reduce the impact associated with poultry production [1]. Soybean meal, as a protein source, stands as one of the most frequently utilized ingredients in livestock feed formulations in the European Union. However, it is noteworthy that its production and transportation from distant producer countries may have substantial environmental repercussions [2]. It should be noted that 97% of the soybean used for animal feed in the EU is imported [3], and that global imports of soybean grain plus soybean meal in the EU are very high, at approximately 30 million tons per year [4]. This heavy reliance on imports makes the EU dependent on other countries and immersed in a vulnerable market, which jeopardizes its sustainability. One practice that could help to decrease this high dependence and lead to greater sustainability may involve the exploration of alternative and locally sourced ingredients with the potential to offer a sustainable and secure supply of nutrition for livestock production [5]. Moreover, there are potential alternative plant ingredients available on the market that remain underutilized in traditional poultry feeding due to their potential anti-nutritional factors or high fiber content. These alternatives include legumes other than soybeans, cereal by-products, and oilseed meals, which could have a considerable protein content [6]. Along these lines, peas, distillers’ dried grains with solubles (DDGs), and sunflower meal can be considered as alternatives. Peas provide both energy and protein in diets, being rich in lysine but with a limited content of sulfur amino acids and tryptophan. In addition, peas may contain anti-nutritional factors (protease inhibitors, lectins, and tannins), although at lower levels than other legumes [7]. DDGs are cereal by-products that contain higher levels of crude protein, fat, minerals, and non-starch polysaccharides (NSPs) than the grain from which this ingredient is derived. However, NSPs are not efficiently digested by monogastrics, so their incorporation may require supplementation with NSP-hydrolyzing enzymes, and they should be used at limited levels [8]. Moreover, sunflower meal has been proposed as a protein alternative in laying hens’ feed, although its use may be limited by its low lysine content and high fiber levels [9]. Moreover, the combination of these ingredients should be studied, since it could have other limiting effects on incorporation or unexpected effects different from those determined individually [10], both on the performance of monogastrics and on the quality of animal products.

In addition to plant-based alternative ingredients, some insect species are considered promising alternatives to traditional sources of protein and fat, due to their nutritional value and lower environmental impact [11], as well as their ability to grow in waste-based substrates [12,13]. This interest is supported by the recent approval of the use of meals made of seven insect species following the introduction of Regulation (EU) 2021/1372 of 17 August [14], which expands their use to include poultry and pigs, in addition to fish. The larvae of one of these species, the black soldier fly (*Hermetia illucens*, Linnaeus, 1758), exhibit the ability to convert organic matter into edible protein and fat during their growth [15]. This species can reach a protein content ranging from 32% to 53% and is considered a viable alternative to conventional protein sources in poultry feed, with the potential to replace ingredients such as soybean meal and fish meal [16]. Most previous research has indicated that insect meals can be viable protein sources without compromising animals´ health or products’ quality [17,18,19]. Moreover, some studies have pointed out the notable interest in the use of live larvae as an ingredient in poultry feed to enhance the welfare of poultry species [20,21,22], as the ingestion of insects is a part of their natural behavior [23]. However, the use of whole dry larvae in poultry feed has not yet been authorized within the European Union (EU)’s regulatory framework. Historically, the EU has required extensive research to ensure the safety and efficacy of novel feed ingredients, as evidenced by the rigorous evaluation process for the approval of insect meal. In this context, despite the evident advantages of using dried larvae, such as the transportation efficiency and ease of storage compared with live insects, authorization is still pending; therefore, studies on this topic are needed to ensure animals’ health and welfare, as well as consumers’ safety.

Given the abovementioned background, our study hypothesized that substituting soybean meal with a combination of peas, DDGs, and sunflower meal and incorporating whole dry larvae of *Hermetia illucens* could enhance the flexibility of diet formulations for laying hens without adversely affecting their performance or egg quality. Consequently, the current study aimed to assess the effects of partly replacing soybean meal with a combination of locally sourced alternative plant-based ingredients and whole dry black soldier fly larvae on the production performance, biochemical blood status, and egg quality of hens.

## 2. Materials and Methods

### 2.1. Ethical Statements

This animal study was carried out in accordance with the protocol approved by the Ethical Committee of Animal Experimentation of the University of Murcia and the Responsible Administrative Institution (Consejería de Agricultura) of the Region of Murcia (Spain) (Ref. No. A12230109), following the European Union’s strategies for the use and care of experimental animals (Directive 2010/63/EU of the EU Parliament and of the Council of 22 September 2010).

### 2.2. Experimental Animals and Treatment Groups

This study was conducted on the experimental farm of the Veterinary Faculty of the University of Murcia (Spain). In total, 120 Isazul layer hens were used in this study. The Isazul breed, characterized as a robust light laying hen, produces an average of 299 cream-colored eggs per year, and is well-adapted to the climatology of southeastern Spain. The hens were sourced from a commercial farm at 17 weeks old (Granja Santa Isabel, Córdoba, Spain). Each hen was weighed and grouped into 15 floor pens (8 hens per pen) to ensure a uniform weight distribution. Each pen (100 cm × 100 cm × 300 cm) included two water dispensers, a central feeder, a nest, perches of sufficient length for resting, and a floor made from a mixture of soil and straw. The study began when all replicates started egg production (23 weeks of age). Subsequently, all pens were randomly assigned to one of the three dietary treatment groups, with five replicates per treatment. The experimental trial lasted for 15 weeks and was split into three experimental sub-periods (Period 1: 23 to 27 weeks of age; Period 2: 27 to 32 weeks of age; Period 3: 32 to 38 weeks of age). All the hens were maintained under consistent husbandry management, including feed and water ad libitum, a 16:8 h light–dark photoperiod, and ambient temperature conditions (13.2–18.2 °C) and relative humidity (63.1–69.0% RH).

### 2.3. Experimental Diets

Three experimental diets were used in this study. A standard soybean–corn–wheat feed was formulated as the control treatment (CON). A second feed was labeled the alternative (ALT) feed, in which the soybean meal and corn (imported ingredients) were partly substituted with alternative plant-based ingredients of local origin, including pea meal, corn DDGs, and a higher proportion of sunflower meal. Additionally, as the third dietary treatment, the ALT feed was supplemented with 5% whole dehydrated black soldier fly larvae (DBSFL), calculated weekly on the basis of the total intake of dry matter from each previous week (ALT+DBSFL). The larvae were supplied by Entomo Consulting S.L. (Cehegín, Murcia, Spain). The CON and ALT diets were formulated to be isoenergetic and isonitrogenous to meet or exceed the requirements for laying hens, according to the recommendations of the Spanish Foundation for the Development of Animal Nutrition (FEDNA) [24]; however, the ALT+DBSFL diet was not isoenergetic or isonitrogenous due to the addition of the larvae. The feeds were presented in pellet format. Detailed information about the diets’ composition is available in Table 1. The analyzed chemical composition of the diets, including the supplied larvae, is presented in Table 2. The DBSFL was provided to the ALT+DBSFL group once a day at the same hour (10:00 am), with the amount calculated as indicated above, in an additional metallic feeder (30 cm Ø). The two remaining replicates of the CON and ALT groups were offered similar but empty feeders to avoid any effect of management on the results.

### 2.4. Hen Performance

The hens were individually weighed using an electronic balance (KERN FKB 8K0.1, KERN & Sohn GmbH, Ziegelei, Balingen, Germany) at the beginning (23 weeks) and in weeks 27, 32, and 38 of the experimental period. The diets were replenished periodically to allow ad libitum feeding. Egg numbers were recorded daily, and weight was documented once a week using an electronic balance (KERN PES 2200-2M, KERN & SOHN GmbH, Balingen, Germany; precision of 0.01 g). Egg production was estimated on basis of the number of eggs and the number of hens present in each cage. The calculation of egg mass per hen was derived by multiplying the daily number of eggs by the corresponding egg weight and dividing this by the number of hens in each treatment. From the data on feed intake and egg mass, the feed conversion ratio (FCR) was calculated. The formulas are as follows:

Egg mass per hen per day = (daily egg number × average egg weight (g))/n of hens

FCR (feed-to-egg-mass ratio) = feed intake (kg)/egg Mass (kg)

### 2.5. Egg Quality Analysis

Throughout the 15-week feeding trial, the measurements of egg quality were determined at 27, 32, and 38 weeks of age. On the day of the test, 4 eggs per replicate (20 eggs per treatment) were selected at random and subjected to internal and external quality assessments within 4 h of collection. Subsequently, under room temperature conditions (18 °C ± 2 °C), each egg was weighed, followed by measurement of the length and width utilizing a 150 mm electronic vernier caliper with a precision of 0.01 mm (Workzone, ALDI Einkauf GmbH & Co. OHG, Essen, Germany). The calculation of the egg shape index was based on the following formula:

Egg shape index = (width/length) × 100

Each egg was then manually broken. Each eggshell’s thickness was measured in three distinct regions of the equator using a specific digital micrometer with a precision of 0.01 mm (Baxlo 3001dig–H, BAXLO, Barcelona, Spain). The resulting values were averaged to obtain the final value in millimeters. Next, the eggshells were carefully cleansed using distilled water and dried in an air-drying oven for 48 h (60 °C). Subsequent weighing was performed using the same digital scale as that used for weighing the eggs. Each broken egg was subjected to measurement of the albumen’s height using an electronic micrometer with a precision of 0.01 mm (Baxlo micrometer in Haugh units, BAXLO, Barcelona, Spain), and the Haugh units were calculated via the following formula:

Haugh Unit = 100 × log (H + 7.57 − 1.7 × W ^0.37^),

where H represents the albumen’s height in millimeters and W represents the egg’s weight in grams [25].

The yolk was separated using a metallic separator and weighed. The albumen’s weight was determined by subtracting the weights of the yolk and dry eggshell from the initial weight of the whole egg. The relative weight of the albumen and yolk with respect to the intact egg’s weight was then calculated. In addition, the yolk color was scored using a 16-point scale by DSM YolkFan™ (DSM-Firmenich, Heerlen, Netherlands). On the next day, four eggs per pen were collected and analyzed to determine the eggshells’ breaking strength (g) using a Brookfield^®^ CT3 Texture Analyzer with TexturePro CT V1.8 Build 31 software (Brookfield Engineering Laboratories, Middleboro, MA, USA).

### 2.6. Blood Status

On the last day of each experimental sub-period (weeks 27, 32, and 38), blood samples were collected from the brachial veins of the wings of two randomly selected animals per replicate (10 for each treatment). These samples were distributed into aliquot tubes with heparin (Vacuette^®^ 4 mL LH Lithium Heparin tubes). The samples were immediately transported to the Interdisciplinary Laboratory of Clinical Analysis of the University of Murcia (Interlab-UMU, Murcia, Spain) for analysis. Plasma isolation was achieved via centrifugation of the samples at 3000 g for 10 min at 4 °C and the samples were preserved at −20 °C, until biochemical analysis, which included quantification of plasma glucose, albumin, total proteins, cholesterol, triglycerides, and uric acid. The plasma’s biochemical parameters were quantified using an automated chemiluminescent immunoassay (Immulite System, Siemens Health Diagnostics, Deerfield, IL, USA).

### 2.7. Chemical Analysis

The experimental diets and DBSFL were ground and sieved through a 0.5 mm sieve using a laboratory mill (RETSCH ZM 200 Ultra Centrifugal Mill; RETSCH, Hann, Germany) and analyzed according the procedures of the Association of Official Analytical Chemists to determine the dry matter (DM) (934.01), crude protein (CP) (2001.11), ether extract (EE) (920.39), and crude fiber (Weende method, 978.10) contents [26]. The ash content was determined by incinerating the samples at 550 °C for 4 h in a muffle furnace (Hobersal HK-11, Barcelona, Spain) following the AOAC 942.05 method [26]. The resulting ashes were dissolved in 50 mL of 0.6 N nitric acid and then filtered. The phosphorus content was determined using the official vanadate–molybdate method, as outlined in the official analytical method indicated by the Commission Regulation (EC) No. 152/2009 [27]. The calcium content was analyzed using atomic absorption spectroscopy (Solaar M Series, Unicam, Cambridge, UK). The starch content was measured using polarimetry according to the official Spanish analytical procedure [28]. To analyze the amino acid content, the samples of the feeds and DBSFL were subjected to a 22 h hydrolysis process in 6 M HCl at 112 °C under a nitrogen atmosphere. Before acid hydrolysis, performic acid oxidation was carried out for methionine and cysteine. The amino acids in the hydrolysate were quantified using high-performance liquid chromatography (HPLC) following post-column derivatization, based on the method outlined by Madrid et al. [29]. Tryptophan was not analyzed.

### 2.8. Statistical Analysis

The obtained data were subjected to analysis using IBM SPSS Statistics 28.0 software (IBM Corporation, Armonk, NY, USA). For the performance parameters and egg quality indicators, each pen was considered an experimental unit, while for the plasma parameters, the experimental unit was the animal. The Shapiro–Wilk test was used to estimate the normality of the data. For normally distributed data (productive performance and egg quality parameters), one-way ANOVA was carried out according to the following model: Yij = µ + Di + eij, where Y is one observation, µ stands for the general mean, D represents the dietary effect, and e represents the error term, for each sub-period and overall. This was followed by Tukey’s post hoc test. For non-normally distributed data (plasma parameters), the Kruskal–Wallis test was carried out. When significant differences were found, a Mann–Whitney U-test for post hoc analysis was carried out. Statistical significance was established at *p* < 0.05.

## 3. Results

### 3.1. Hens’ Performance

The performance parameters of the laying hens across the three experimental sub-periods (23 to 27, 27 to 32, and 32 to 38 weeks of age) and overall (23 to 38 weeks of age) are presented in Table 3. There was no significant difference in body weight across the three treatments, regardless of the time frame analyzed or whether all the sub-periods were examined together. The feed intake among the treatment groups was similar for both the periodic and the overall measurements (*p* > 0.05).

There was no difference in egg production across the dietary treatments, regardless of the period examined (*p* > 0.05). In addition, the egg weight and egg mass were not affected by the dietary treatments (*p* > 0.05). The FCR (expressed as the feed-to-egg-mass ratio) was also similar among the dietary treatments across all the experimental periods (*p* > 0.05).

### 3.2. Egg Quality

The impact of the different dietary treatments on the egg quality of the laying hens is presented in Table 4 for each experimental sub-period (23 to 27, 27 to 32, and 32 to 38 weeks of age) and overall. The egg shape index did not show any statistical differences across the three treatments, regardless of the time frame analyzed, while the Haugh units only showed significant differences in the first sub-period (23–27 weeks), with the Haugh units of the eggs from the hens fed the ALT+DBSFL diet being higher than those from the hens fed the ALT diet (*p* < 0.05), but no differences were observed with respect to the CON diet. There were no significant differences in the albumen and yolk percentages or shell characteristics across the treatments. With respect to the color of the yolk, no differences were observed (*p* > 0.05).

### 3.3. Biochemical Plasma Profile

Table 5 reports the biochemical profiles of the laying hens according to the dietary treatments. The diets had no significant effect on the plasma glucose, albumin, total protein, or uric acid contents of the hens across the experimental period. Supplementation with whole DBSFL decreased the plasma cholesterol and triglycerides of the hens compared with the other two dietary treatments only during the second experimental sub-period (32 weeks of age) (*p* < 0.05). There were no statistical differences between the CON and ALT diets.

## 4. Discussion

The use of alternative protein sources in poultry feed offers a promising approach to alleviating the high dependence on imported ingredients and environmental pressures linked to the current feeding practices in the European Union, with the potential to improve the prospects of production in the current context. Therefore, the aim of our study was to evaluate the effects on the production performance, egg quality, and blood parameters of laying hens via the partial substitution of imported ingredients such as soybean meal with alternative options. These alternatives, which are common in feed manufacturing but less typical in layer hens’ diets, include a combination of peas, corn DDGs, and a higher proportion of sunflower meal.

Notably, combinations of alternative plant-based ingredients should be studied because their limitations are known individually, but their combined administration could reveal new and unexpected effects. There are few studies in the literature on the effect of combinations of alternative plant-based ingredients on monogastric performance [10,30]. It is worth noting that in this study, an alternative diet with such plant-based ingredients (ALT) was formulated to be isoenergetic and isonitrogenous with respect to the control diet (CON), showing a similar analytical composition with very slight differences, as expected.

Furthermore, our research explored the effects of supplementing layer hens’ diet with 5% whole DBSFL, which contains high levels of crude fat (31.58% on a DM basis) and crude protein (36.31% on a DM basis), with an amino acid profile of 2.29% lysine and 1.76% methionine (on a DM basis). These levels are in accordance with the range described by Lu et al. [16] for full-fat larvae. Thus, the ALT+DBSFL diet was richer in protein and energy compared to the CON and ALT diets. This novel ingredient is currently being investigated for application in poultry feeding, as its protein can be efficiently utilized and is, therefore, considered a potential alternative to traditional protein sources such as soybean or fish meal [31,32]. However, its use as dried whole larvae has not yet been authorized, and further studies are needed to prove its safety in animals and their products with a view toward its approval.

Our study revealed that replacing imported ingredients using a combination of locally sourced alternative plant-based ingredients and adding 5% whole DBSFL to the diet did not adversely affect body weight. In this vein, Ciurescu and Pana [33] did not observe any negative effects on body weight when including peas at 35% (with or without enzymes) in the diet of laying hens between 20 and 54 weeks of age. Moreover, Shi et al. [34] incorporated sunflower meal at levels of 8.26%, 16.52%, and 24.84% into the diets of hens at 28 to 34 weeks of age to replace soybean meal, observing no significant effect on weight gain. Pirgozliev et al. [35] assessed the partial replacement of soybean in the diets of laying hens at the end of the production cycle (included as a feed with 7.5% soybean meal and 5% whole soybean) using a combination of sunflower meal at 7.5% and rapeseed meal at 7%. Their findings indicated that this substitution did not adversely affect the body weights of the animals.

In agreement with our results obtained using the ALT+DBSFL diet, Ruhnke et al. [36], in a free choice feeding trial offering whole black soldier fly larvae (BSFL) ad libitum to laying hens (resulting in a 16% contribution to the total intake), reported no significant effects on the body weights of the birds. Similarly, Kawasaki et al. [37] found no significant impact on body weight when administering a diet containing 10% whole non-defatted BSFL to laying hens. Additionally, other studies have incorporated insects, either totally or partly defatted, into hens’ diets. Thus, Maurer et al. [38] observed no effects on the body weights of hens when partly or fully replacing soybean cake with BSFL meal (partly defatted), even at the high inclusion levels of 12% or 24%. However, it is worth noting that this experiment was only conducted over a 3-week feeding period. Conversely, Marono et al. [17] reported that when defatted BSFL meal was used as a total replacement for soybean meal, the body weights of laying hens at 45 weeks of age were negatively impacted.

In accordance with the body weights of hens, feed intake was not affected by the diet containing corn DDGs, peas, and a higher proportion of sunflower meal. Ghazalah et al. [39] reported no differences in feed consumption when corn DDGs were included in a proportion of up to 23.17% in the diet of laying hens. Regarding peas, Fru-Nji et al. [40] observed that increasing their inclusion up to 50% in layers’ diets increased the animals’ feed consumption. However, both studies indicated that other performance parameters could be negatively affected by increasing the levels of DDGs or peas. Moreover, Shi et al. [34] indicated that including up to 24.84% of sunflower meal in layers’ diets did not affect the feed intake. Similarly, in this study, supplementing with 5% whole DBSFL did not influence total intake. It might be expected that the consumption of the insect-supplemented diet would be lower compared with non-supplemented diets, given the high crude fat content of insects, which increases the energy concentration of the diet and could consequently reduce intake. However, Liu et al. [41] found that offering different percentages of whole BSFL meal in the feed resulted in a higher feed intake at the 3% inclusion level, and they attributed this phenomenon to the increased palatability. In addition, Bejaei and Cheng [42] observed no effects on feed intake when replacing 50% of soybean meal and 90% of soybean oil with chopped whole larvae in diets for hens. Similarly, Ruhnke et al. [36] reported no significant impact on feed intake when offering whole black soldier fly larvae (BSFL) ad libitum to laying hens. In contrast, another study using defatted BSFL meal showed a significant reduction in intake of 13.6% when insects were included at 17% in the feed [17].

In our experiment, we observed no effects of the ALT diet on egg production, egg weight, egg mass, or FCR. These results agree with the results reported by Fru-Nji et al. [40], who found no negative consequences of incorporating 0–50% peas in the feed on the egg production, egg mass, and feed conversion of laying hens between 24 and 72 weeks of age. Additionally, Ciurescu and Pana [33] reported that incorporating peas at 35% as a partial substitute for soybean meal in the feed of laying hens, along with the inclusion of the enzymes protease and cellulase, either alone or in combination with pectinase, xylanase, glucanase, and α-amylase, could increase the laying rate (by 1.4 to 3.0%) and egg mass (by 1.7 to 3.4%). The incorporation of corn DDGs in layers’ feeds has also been studied. A decline in laying percentage, egg mass, and feed conversion ratio was observed at the level of 7.72% in the diet without enzymes; although when enzymes (glucanase, xylanase, amylase, polygalacturonase, and protease) were added, this decline was only observed at a higher level, of 15.45% [39]. The effects of including sunflower meal in the diet of layers on the performance parameters have yielded variable results. However, it has been reported that when diets are properly formulated to correct for deficiencies in amino acids and energy, even common sunflower meals can be used without penalizing the production rates of laying hens [43]. Thus, de Morais Oliveira et al. [44] evaluated the performance of hens in a semi-intensive system that were fed different levels of sunflower meal with a low crude protein content (25.71%) in the diet (4.45%, 8.89%, and 13.34%) to replace soybean meal, determining that there were no negative effects on egg production, egg weight, egg mass, or FCR. Furthermore, Saleh et al. [9] included a high-quality sunflower meal with a high crude protein content (36%) in a proportion of up to 10% in the diet of hens in the late phase of laying and found beneficial effects on egg production and FCR.

The inclusion of a combination of less common plant-based ingredients in laying hens’ feed, even at acceptable levels, can trigger unpredicted effects. Rama Rao et al. [10] evaluated the effects of including a mixture of alternative vegetable protein ingredients partly or totally substituting for soybean meal in the diet of laying hens. They observed that this strategy did not affect egg production and FCR when up to 16% of the diet included these ingredients (9.0% cottonseed, 4.5% DDGs, and 2.5% rapeseed meal). However, at higher levels (20% of the mixture), egg mass, intake, and FCR were impaired, even when the recommended levels of inclusion for the individual ingredients were followed. In our experiment, the combination of peas, DDGs, and sunflower meal at 19% in the diet did not harm the production performance of the hens.

Regarding the supplementation with insects in our trial, no negative effects on egg production, egg weight, egg mass, or FCR were observed, which is in line with the results obtained by Ruhnke et al. [36], who offered whole BSFL ad libitum to laying hens and found no effect on egg production, egg mass, or FCR. Moreover, Bejaei and Cheng [42] did not find effects on egg production when partly replacing soybean meal with 10% full-fat BSFFL. However, in another study, Bejaei and Cheng [45] found that incorporating 10–18% full-fat dry black soldier fly larvae (DBSFL) into hens’ diets as a partial or total replacement for soybean meal resulted in lighter eggs compared with the control eggs. Similarly, Ruhnke et al. [36], who offered whole dehydrated larvae ad libitum to hens, observed a decrease in egg weight relative to the controls. By contrast, Liu et al. [41] incorporated BSFL meal in the diet of hens between 45 and 53 weeks of age, and they found an improvement in egg weight with the inclusion of 3% and in FCR with the inclusion of 5% when evaluated over the entire experimental period, compared with the control diet. Conversely, Marono et al. [17] observed a decrease in egg production, egg weight, and egg mass in laying hens between 24 and 45 weeks of age when they completely replaced soybean meal with 17% defatted BSFL in the diet. These negative effects were consistent with a decrease in the intake of the insect meal treatment, despite the diet being formulated to be isoproteic and isoenergetic compared with the control. In contrast, Bovera et al. [46] reported increased egg mass when they included partly defatted BSFL meal at 7.3% and 14.6% in the diet to replace 25% and 50% of the soybean meal, respectively, without negatively affecting other performance parameters. Certainly, the lack of differences in performance parameters between dietary groups further confirmed that soybean meal can at least be partly substituted by alternative plant-based ingredients and supplemented with whole DBSFL without compromising productive parameters. This indicates that the administered diets are sufficiently palatable and that there is comparable dietary effectiveness across all groups. Notably, although we did not find statistical differences in the performance parameters when the larvae were included, we observed quantitative improvements in egg production, egg weight, egg mass, and FCR in comparison with the other treatments.

In this research, the inclusion of alternative plant-based ingredients did not affect the quality of the eggs in relation to the morphometric parameters and structure of the eggs’ components. In general, Ghazalah et al. [39] found no difference in the egg shape indexes, Haugh units, and thicknesses of the shells in the eggs of layers when incorporating corn DDGs in a proportion of up to 23.17% in the diet (with or without enzyme supplementation). However, the eggshells’ weights decreased when the incorporation of DDGs was 15.45% of the diet. Similarly, Ciurescu and Pana [33] did not find negative effects on the internal and external traits of egg quality when incorporating peas at 35% (with or without enzymes) in layers’ diet, aligning with our findings. Additionally, Shi et al. [34] incorporated sunflower meal at levels up to 24.84% in hens’ diets to replace soybean meal, and observed no significant effects on Haugh units, percentage of albumen, percentage of yolk, percentage of shell, the shells’ thickness, or the shells’ strength. These results are consistent with our findings, despite our use of a lower level of sunflower meal compared with the maximum level used by these authors. Regarding the incorporation of combinations of plant-based protein ingredients, we observed variable effects on egg quality. Yildiz et al. [47] reported that including sunflower meal at 5% and corn DDGs at 10%, 20%, or 30% as partial substitutes for soybean meal in the diet of layer hens (from 32 to 46 weeks of age) did not diminish traits of egg quality such as Haugh units, the shells’ thickness, and the shells’ strength; these results are in line with our findings. Other combinations have also been evaluated, showing no significant effects on the characteristics of egg quality, as reported in the study by Rama Rao et al. [10], which used cotton meal, DDGs, and mustard seed meal, or the study by Pirgozliev et al. [35], which included sunflower and rapeseed meal. However, some combinations have shown effects on the internal or external quality of eggs, such as the incorporation of a combination of yellow lupins, narrow-leaved lupins, and peas in layers’ diet by Kowalska et al. [48], or the use of low-gossypol cottonseed meal and double-zero rapeseed meal by Wang et al. [49]. These findings underscore the importance of evaluating the types of vegetable sources, their level of incorporation, and the combination of ingredients in the diet, as they can affect not only the performance parameters but also the external and internal quality of eggs.

In our experiment, the inclusion of alternative plant-based ingredients with DBSFL supplementation had minimal effects on the quality of eggs produced by hens with respect to the morphometric parameters and structure of the eggs. However, other studies administering whole larvae, such as that of Ruhnke et al. [36], observed a decrease in the weight and thickness of shells in eggs from hens fed whole dry black soldier fly larvae (DBSFL) ad libitum. In addition, Bejaei and Cheng et al. [45] observed that incorporating 10–18% full-fat DBSFL into hens’ diets as a partial or total replacement for soybean meal resulted in lower weights of the shell and albumen compared with the control eggs. However, in line with our results, Star et al. [20] added live BSFL (at 2.83% of DM) to a feed that completely replaced soybean meal with alternative protein ingredients, such as rapeseed meal, gluten meal, and potato protein, and observed no effects on the Haugh units or the eggshells’ strength. In another study, Park et al. [50] found no differences in Haugh units or the shells’ thickness and strength when defatted larvae meal was included in the diet at 2% or 4%. However, the data from the present experiment indicated significant effects on the Haugh units at 27 weeks of age, with higher levels observed when the ALT+DBSFL diet was administered compared with the ALT diet. These results align with those of Liu et al. [41], who also reported higher Haugh units in the early stages of the trial with laying hens (at 49 weeks of age) when supplemented with 3% BSFL meal compared with the control diet. However, similar to our results, these differences were not observed during the later phases of the experiment (at 53 weeks of age). It should be noted that Haugh units are important indicators of the internal quality of fresh eggs, but this measurement is influenced by various factors, such as the age and genotype of the hens, molting, environmental conditions, nutrition, and the storage conditions of the eggs [51].

These findings contribute to the growing body of evidence supporting the potential of alternative plant-based protein sources, in combination with whole dry black soldier fly larvae, as substitutes for or complements to imported ingredients within the nutrition of egg-laying poultry.

The yolk color was not affected by the dietary treatments in our experiment. In contrast, Ghazalah et al. [39] observed that increasing corn DDGs in the layers’ diet (7.72%, 15.45%, and 23.17%) increased the egg yolks’ color score. Additionally, an increase in yolk color was described by Ciurescu and Pana [33] when the proportion of peas was increased to 35%. Regarding the inclusion of sunflower meal in hens’ diets, different effects have been found. While Saleh et al. [9] observed an improvement in yolk color when 5% or 10% of this ingredient was incorporated, Shi et al. [34] reported no significant effects on this parameter when sunflower meal was included at levels up to 24.84%. These ingredients have natural pigments, although the effect on yolk color is not always achieved, since this effect depends on the levels of incorporation, the types of pigment, the interaction with the rest of the ingredients or components in the diet, and even the health and age of the hens [52,53,54]. Secci et al. [55] found that supplementation with defatted BSFL meal in the diet of laying hens intensified the color of the yolk; they justified this finding as being caused by the content of pigments available in the insect meal, as well as the increase in the percentage of corn in the diet. However, in our case, the corn content of the ALT and ALT+DBSFL diets was lower than that of the control diet. Despite this, the yolk color was maintained.

When the impact on the health status of the laying hens was studied, we observed that the inclusion of alternative plant-based ingredients did not influence the blood chemistry indices evaluated. Other authors have found different effects when incorporating legumes into layers’ diets. For instance, Straková et al. [56] reported changes in plasma glucose, total protein, cholesterol, and triglyceride concentrations, but not uric acid, when partly replacing soybean meal (50%) with lupin meal (hulled or dehulled) in a long-term experiment on layer hens; they noticed increased glucose levels and decreased levels of the other metabolites. However, Moschini et al. [57], using peas (at 35.3% or 17.6%), faba beans (at 9.2% or 24.6%), or lupin (at 32.0% or 19.0%) to partly substitute for soybean meal, found no effects on the concentration of total protein or albumin in broilers’ plasma, which aligns with our results. The effects of legume seeds on the digestion and metabolism of nutrients can be variable and may influence blood’s biochemical status to different degrees [58]. Regarding the inclusion of sunflower meal, Sherif et al. [59] indicated that inclusion of up to 27% in the diet did not affect glucose, total protein, or cholesterol levels in the sera of laying hens. Additionally, Abd El-Hack et al. [60] found that incorporating DDGs at levels of 5% or higher in hens’ diets increased total cholesterol and triglycerides, although these effects were not observed in our study.

It should be noted that when we supplemented the ALT diet with whole DBSFL, only the levels of total cholesterol and triglycerides in the blood plasma of the hens decreased at 32 weeks of age. In this regard, Bejaei and Cheng [42] observed a reduction in plasma triglycerides in laying hens when incorporating 18% full-fat dry black soldier fly larvae (DBSFL) into the diet. However, this effect was not observed when DBSFL was incorporated at 10%. On the other hand, Bovera et al. [46] and Marono et al. [17] also reported decreases in cholesterol and triglyceride levels when partly or totally defatted BSFL was included in the diet to substitute for soybean meal (partly or totally, respectively). This effect could have been due to the insect’s chitin content. Prajapati and Patel [61] indicated that chitin could reduce the absorption of lipids in the intestine by attracting fatty acids and bile acids due to their different charges, which could result in lower lipid levels in the blood. Following the same reasoning, Gariglio et al. [62] also reported lower levels of total cholesterol and triglycerides in Muscovy ducks when incorporating partly defatted BSFL meal in their diets; the authors attributed this finding to the presence of chitin in BSFL. Moreover, Bongiorno et al. [63] found that the cholesterol levels of medium-growing chickens were lower in the groups administered live BSFL compared with the control group, and also attributed this effect to the chitin content of the larvae. In contrast, other studies did not find changes in cholesterol or triglyceride profiles when substituting soybean meal with defatted BSFL at 5%, 10%, and 15% in the diet [64]. Attivi et al. [65] reported increases in the plasma levels of cholesterol and triglycerides when whole BSFL was included in the diet (at 6% and 8%, respectively) of 40-week-old laying hens. Although these authors formulated isoenergetic diets, the crude fat content increased as the level of whole BSFL included in the diet increased. In our work, despite including whole larvae as a feed additive, which increased the fat intake of the hens, no increase in cholesterol or triglycerides was observed. On the other hand, Fiorilla et al. [66] showed that slow-growing chickens fed either live or dehydrated black soldier fly larvae exhibited increased chitinase activity in the proventriculus, suggesting the improved digestibility of insect-derived bio-compounds.

The absence of significant variations in the main plasma biochemical parameters across the three dietary groups suggested that the partial substitution of the primary imported ingredients with alternative plant-based protein sources and the inclusion of whole DBSFL had no adverse impact on the health status of laying hens.

## 5. Conclusions

The conclusions derived from this study suggest that including a 19% mix of plant-based alternative ingredients (peas, DDGs, and sunflower meal) in the diet to partially replace soybean meal did not negatively affect the hens’ weight or production performance indicators during laying (production percentage, egg weight, egg mass, and feed conversion efficiency). These parameters were not compromised by supplementation with whole DBSFL at the levels tested. Additionally, the external and internal egg qualities, as well as the plasma biochemical profiles of the hens, were not adversely affected by the use of alternative plant-based ingredients or the inclusion of larvae. Thus, the proposed feeding strategies are of great interest for improving the sustainability of egg production and decreasing the dependence on imported ingredients. Moreover, these findings contribute to the growing body of evidence supporting the safety of incorporating whole dried insects into poultry feed, as it does not affect the performance, product quality, or blood parameters. These findings can inform the regulatory approval process within the European legislative framework. It should be noted that further research is necessary to evaluate these alternative ingredients in layers’ diets—either alone or in combination—in order to develop a flexible formulation that guarantees productivity, sustainability, egg quality, and hen health. However, further research may be required to evaluate the impact of these diets throughout the entire production cycle of laying hens and on a commercial scale.

## Figures and Tables

**Table 1:** Ingredients and calculated composition of the two experimental diets (percentage of as-fed basis).

	CON ^1^	ALT ^2^
Ingredients		
Corn	41.58	35.29
Soybean meal (46% crude protein)	21.97	15.00
Wheat	14.00	14.68
Calcium carbonate	8.69	8.90
Corn DDGs ^3^	-	7.46
Soybean hulls	3.05	-
Sunflower meal (28% crude protein)	2.50	6.00
Peas	-	5.56
Soybean oil	2.50	2.50
Barley	2.00	2.50
Wheat middling	1.67	0.09
Monocalcium phosphate	0.67	0.52
Diatomaceous earth	0.50	0.50
Premix ^4^	0.33	0.33
Sodium chloride	0.25	0.20
DL–methionine (99%)	0.19	0.18
Sodium bicarbonate	0.07	0.12
L–lysine 50	0.03	0.16
Calculated composition ^5^		
AMEn (MJ/kg) ^6^	11.43	11.43
Crude protein (%)	16.4	16.4
Lysine (%)	0.83	0.83
Methionine (%)	0.45	0.45
Methionine+cysteine (%)	0.73	0.73

^1^ CON: control diet. ^2^ ALT: alternative diet with soybean meal partly replaced with alternative plant ingredients. ^3^ DDGs, dried distillers’ grains with solubles. ^4^ Premix provided per kilogram of feed: Vitamin A, 7500 IU; Vitamin D3, 1500 mg; Vitamin K3, 1.5 mg; Vitamin B2, 3 mg; Vitamin B12, 10 µg; nicotinamide, 15 mg; D-calcium pantothenate, 7 mg; pantothenic acid, 6.44 mg; betaine, 54.15 mg; choline chloride, 127.5 mg; Fe, 18 mg as ferrous sulfate monohydrate; Cu, 4 mg as copper sulfate pentahydrate; Zn, 37 mg as zinc oxide; Mn, 65 mg as manganese (II) oxide; I, 1.9 mg as potassium iodate; selenium, 0.1 mg as sodium selenite; 600 FTU of 6-phytase EC 3.1.3.26 (1 FTU is the amount of enzyme that liberates 1 micromole of inorganic phosphate per minute from sodium phytate at pH 5.5 and 37 °C); and 1500 EPU of endo-1,4-β-xylanase EC 3.2.1.8 (1 EPU is the amount of enzyme that liberates 0.0083 micromoles of reducing sugars (xylose equivalents) from oat spelt xylan per minute at pH 4.7 and 30 °C). ^5^ According to the Fundación Española para el Desarrollo de la Nutrición Animal (FEDNA, 2018). ^6^ AMEn: apparent metabolizable energy corrected for nitrogen retention.

**Table 2:** Chemical composition of the experimental diets and dry black soldier fly larvae used in the study (percentage of as-fed basis).

	CON ^1^	ALT ^2^	DBSFL ^3^
Dry matter	90.9	91.2	96.1
Crude protein	16.1	16.2	34.9
Ether extract	7.80	7.10	30.3
Crude fiber	6.90	6.30	7.75
Ash	12.0	11.7	11.8
Starch	38.0	36.3	-
Calcium	3.84	3.73	2.89
Total phosphorus	0.595	0.570	0.695
Indispensable amino acid			
Arginine	0.791	0.827	1.46
Histidine	0.294	0.302	0.795
Isoleucine	0.488	0.460	1.43
Leucine	1.00	1.04	2.22
Lysine	0.827	0.848	2.20
Methionine	0.487	0.582	1.69
Methionine + cysteine ^4^	0.886	0.914	2.13
Phenylalanine	0.564	0.569	1.29
Threonine	0.478	0.496	1.28
Valine	0.641	0.704	2.01

^1^ CON: control diet. ^2^ ALT: alternative diet with soybean meal partly replaced by alternative plant ingredients. ^3^ DBSFL: whole dry black soldier fly larvae. ^4^ Cysteine is a semi-essential amino acid.

**Table 3:** Effects of the experimental diets on the performance parameters of laying hens across the three experimental sub-periods and overall.

	CON ^1^	ALT ^2^	ALT+DBSFL ^3^	SEM ^4^	*p*-Value
Body weight (g)					
23 weeks of age (initial)	1873	1882	1886	25.4	0.979
27 weeks of age	2018	2059	2040	30.7	0.860
32 weeks of age	2144	2181	2116	31.4	0.707
38 weeks of age (final)	2240	2296	2181	30.7	0.344
Feed intake (DM) (g/d) ^5^					
23–27 weeks of age	107	112	113	2.04	0.525
27–32 weeks of age	116	117	114	1.93	0.762
32–38 weeks of age	121	122	119	2.12	0.830
Overall period	116	118	115	1.81	0.867
Egg production (%)					
23–27 weeks of age	66.7	64.5	71.3	1.63	0.263
27–32 weeks of age	76.2	76.1	79.7	1.72	0.644
32–38 weeks of age	75.3	74.9	76.7	1.71	0.907
Overall period	73.3	72.5	76.2	1.22	0.452
Egg weight (g)					
23–27 weeks of age	60.8	60.9	62.2	0.65	0.634
27–32 weeks of age	62.9	63.5	63.3	0.45	0.875
32–38 weeks of age	65.9	65.7	66.9	0.59	0.666
Overall period	63.5	63.7	64.4	0.50	0.734
Egg mass (g egg/hen/day)					
23–27 weeks of age	40.2	40.1	43.7	1.13	0.361
27–32 weeks of age	48.0	48.4	50.4	1.26	0.708
32–38 weeks of age	49.6	49.2	51.3	1.24	0.779
Overall period	46.6	46.5	49.0	1.00	0.537
Feed-to-egg-mass ratio (FCR) (kg/kg)					
23–27 weeks of age	2.69	2.82	2.59	0.062	0.343
27–32 weeks of age	2.43	2.44	2.26	0.035	0.101
32–38 weeks of age	2.46	2.48	2.32	0.060	0.515
Overall period	2.49	2.53	2.36	0.032	0.113

^1^ CON: control diet. ^2^ ALT: alternative diet with soybean meal partly replaced by alternative plant ingredients. ^3^ ALT+DBSFL: identical to ALT but with the inclusion of whole dry black soldier fly larvae. ^4^ SEM: standard error of the mean (n = 5 for each treatment. ^5^ Feed intake in the ALT+DBSFL group, including the larvae intake.

**Table 4:** Effects of the experimental diets on the quality of eggs of laying hens across the three experimental sub-periods and overall.

	CON ^1^	ALT ^2^	ALT+DBSFL ^3^	SEM ^4^	*p*-Value
Egg shape index (%)					
23–27 weeks of age	77.2	76.6	77.2	0.383	0.774
27–32 weeks of age	78.4	76.2	76.6	1.111	0.698
32–38 weeks of age	76.3	75.9	76.7	0.488	0.830
Overall period	77.3	76.2	76.8	0.481	0.688
Haugh unit					
23–27 weeks of age	86.3 ^a,b^	84.1 ^b^	91.7 ^a^	0.933	0.017
27–32 weeks of age	84.5	82.1	86.8	1.150	0.494
32–38 weeks of age	84.6	84.7	87.2	1.206	0.615
Overall period	85.0	83.7	88.3	1.020	0.210
Albumen percentage (%)					
23–27 weeks of age	63.7	64.2	63.4	0.175	0.184
27–32 weeks of age	63.2	63.4	62.9	0.210	0.624
32–38 weeks of age	60.9	61.5	61.3	0.293	0.649
Overall period	62.4	62.9	62.4	0.140	0.286
Yolk percentage (%)					
23–27 weeks of age	26.5	26.4	26.9	0.142	0.343
27–32 weeks of age	27.5	27.4	27.8	0.210	0.780
32–38 weeks of age	28.8	28.5	28.7	0.210	0.883
Overall period	27.7	27.6	27.9	0.110	0.498
Dry shell weight (g)					
23–27 weeks of age	5.80	5.74	5.76	0.076	0.955
27–32 weeks of age	6.01	5.97	5.93	0.118	0.963
32–38 weeks of age	6.09	6.11	6.37	0.079	0.286
Overall period	5.98	5.96	6.06	0.062	0.794
Shell thickness (mm)					
23–27 weeks of age	0.430	0.440	0.460	0.007	0.489
27–32 weeks of age	0.440	0.440	0.440	0.005	0.861
32–38 weeks of age	0.480	0.470	0.450	0.005	0.084
Overall period	0.460	0.450	0.450	0.003	0.555
Shell breaking strength (g)					
23–27 weeks of age	4159	4393	4692	207.8	0.590
27–32 weeks of age	4191	4624	4268	144.3	0.451
32–38 weeks of age	4208	4169	4810	197.0	0.362
Overall period	4189	4380	4598	135.7	0.491
Yolk color (DSM scale)					
23–27 weeks of age	5.77	6.60	6.67	0.281	0.377
27–32 weeks of age	8.05	8.50	8.40	0.147	0.446
32–38 weeks of age	8.40	8.85	9.00	0.099	0.072
Overall period	7.58	8.13	8.18	0.124	0.134

^1^ CON: control diet. ^2^ ALT: alternative diet with soybean meal partly replaced by alternative plant ingredients. ^3^ ALT+DBSFL: identical to ALT but with the inclusion of whole dry black soldier fly larvae. ^4^ SEM: standard error of the mean (n = 5 for each treatment. ^a,b^ Different superscripts in the same row mean statistical differences at *p* < 0.05.

**Table 5:** Effects of the experimental diets on the plasma biochemical parameters of laying hens at the end of the three experimental sub-periods.

	CON ^1^	ALT ^2^	ALT+DBSFL ^3^	*p*-Value
Glucose (mg/dL)				
27 weeks	160.20 (89.05–169.50) ^4^	147.65 (120.68–162.90)	170.70 (133.70–180.85)	0.198
32 weeks	160.75 (132.33–171.60)	164.30 (147.10–183.10)	164.20 (137.28–193.63)	0.756
38 weeks	170.65 (147.25–190.83)	172.00 (143.95–183.85)	170.65 (167.50–197.73)	0.642
Albumin (g/dL)				
27 weeks	1.64 (1.52–1.75)	1.56 (151–1.605)	1.60 (1.47–1.70)	0.546
32 weeks	1.68 (1.64–1.72)	1.67 (1.65–1.76)	1.63 (1.58–1.71)	0.439
38 weeks	1.85 (1.81–1.92)	1.82 (1.74–1.85)	1.84 (1.79–1.90)	0.490
Total protein (g/dL)				
23–27 weeks	4.75 (4.46–5.28)	4.17 (3.96–4.49)	4.66 (4.19–4.97)	0.178
27–32 weeks	4.81 (4.70–4.95)	4.89 (4.70–5.12)	4.70 (4.56–4.78)	0.339
32–38 weeks	5.56 (5.38–5.73)	5.32 (5.12–5.53)	5.48 (5.26–5.58)	0.363
Cholesterol (mg/dL)				
27 weeks	102.1 (85.92–188.4)	110.7 (81.5–124.9)	97.66 (74.44–113.1)	0.568
32 weeks	143.1(113.2–160.1) ^a^	145.6 (121.1–193.9) ^a^	112.6 (88.0–120.9) ^b^	0.028
38 weeks	159.1 (133.8–177.1)	165.6 (146.7–202.4)	128.2 (110.3–154.2)	0.112
Triglycerides (mg/dL)				
27 weeks	1289 (847.9–1920)	1337 (954.6–1650)	1134 (789.6–1356)	0.278
32 weeks	1829 (1714–2076) ^a^	1935 (1564–2173) ^a^	1235 (967.1–1649) ^b^	0.027
38 weeks	1786 (1676–1972)	1876 (1660–2048)	1692 (1253–1845)	0.412
Uric acid (mg/dL)				
27 weeks	5.12 (4.79–6.20)	4.00 (2.94–5.84)	5.47 (4.15–6.03)	0.587
32 weeks	3.94 (1.99–4.62)	3.78 (2.14–5.74)	4.77 (3.61–6.13)	0.516
38 weeks	3.58 (1.08–6.81)	3.57 (1.60–5.32)	3.27 (2.18–5.67)	0.939

^1^ CON: control diet. ^2^ ALT: alternative diet with soybean meal partly replaced by alternative plant ingredients. ^3^ ALT+DBSFL: identical to ALT but with the inclusion of whole dry black soldier fly larvae. ^4^ Data are expressed as the median ± range (minimum to maximum). ^a,b^ Different superscripts in the same row mean statistical differences at *p* < 0.05.

## Data Availability

The data presented in this study are available on request from the corresponding author.
